# Intraspecific Variation in Parental Care May Reflect Variation in Parental Quality

**DOI:** 10.1002/ece3.70578

**Published:** 2024-11-13

**Authors:** Natalie Pilakouta, Elizabeth J. H. Hanlon, Per T. Smiseth

**Affiliations:** ^1^ Institute of Ecology and Evolution, School of Biological Sciences University of Edinburgh Edinburgh UK; ^2^ Centre for Biological Diversity, School of Biology University of St Andrews St Andrews UK

**Keywords:** biparental care, burying beetle, life history, *Nicrophorus vespilloides*, reproductive investment, resource acquisition, resource allocation, trade‐offs

## Abstract

The existence of life‐history trade‐offs is a fundamental assumption of evolutionary biology and behavioural ecology, yet empirical studies have found mixed evidence for this. Such trade‐offs are expected when individuals vary in how they allocate their limited resource budgets between different life‐history functions (variation in resource allocation), but they may be masked when individuals vary in how many resources they have acquired that they can later allocate to life‐history functions (variation in resource acquisition). We currently lack studies on the extent to which individual differences in behaviour reflect variation between individuals in resource acquisition and resource allocation. Here, we use parental care as a case study for exploring this question. We used the burying beetle *Nicrophorus vespilloides* , which exhibits facultative biparental care, comprising direct care (provisioning food or interacting with larvae) and indirect care (guarding or maintaining the carcass). We found some evidence for a positive relationship between these two components of care for both male and female parents. In addition, parents that spent more time providing care 24 h after hatching also tended to provide care for longer. Lastly, parents that provided more parental care did not experience a trade‐off of reduced lifespan after the breeding attempt. On the contrary, we found a positive relationship between the duration of care provided and parents' post‐breeding lifespan. Our finding of positive relationships between parental behaviours and between parental care and lifespan suggests that variation in care was mainly driven by differences in prior resource acquisition (i.e., parental quality) among individuals rather than differences in resource allocation. Our findings thus suggest that high intraspecific variation in parental quality can potentially mask reproductive investment trade‐offs within populations.

## Graphical abstract

In this study, we find no evidence for trade‐offs between parental behaviours or between parental care and post‐breeding lifespan. Instead, the positive relationships between these traits suggest that variation in care was mainly driven by individual differences in parental quality. Our results thus indicate that high intraspecific variation in parental quality can mask reproductive investment trade‐offs within populations.

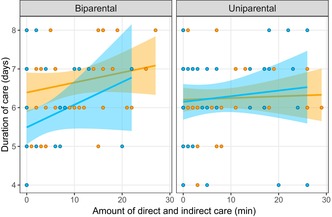

## 1 Introduction

The existence of life‐history trade‐offs between mutually exclusive functions is a fundamental assumption of evolutionary biology and behavioural ecology (Stearns 1977, 1989). For example, evolutionary theory predicts that organisms should face trade‐offs between reproduction and longevity or between the number and size of their offspring (Fisher 1930; Cody 1966; Stearns 1992). Nevertheless, a recent meta‐analysis revealed that empirical studies often find little or no evidence for such trade‐offs at the phenotypic level (Haave‐Audet et al. 2022). Similarly, another meta‐analysis found no evidence for the existence of trade‐offs at the genetic level (Chang et al. 2024). Instead, they found an overall positive genetic correlation between survival and other life‐history traits (Chang et al. 2024).

A potential explanation for the absence of such trade‐offs is that individuals vary not just in how they allocate their limited resource budgets between different functions (i.e., variation in resource allocation), but also in how many resources they have acquired that they later can allocate to life‐history functions (i.e., variation in resource acquisition). Such variation has important implications for our ability to detect life‐history trade‐offs because it generates positive correlations between life‐history functions that may mask any evidence of life‐history trade‐offs at the phenotypic level (van Noordwijk and de Jong 1986; Laskowski, Moiron, and Niemela 2021). Negative correlations between life‐history traits are expected when variation in resource allocation exceeds variation in resource acquisition, while positive correlations are expected when variation in resource acquisition exceeds variation in resource allocation (van Noordwijk and de Jong 1986).

A recent review highlighted the importance of integrating behaviour into life‐history theory by explicitly considering whether differences in behaviour are more closely associated with variance in resource acquisition or resource allocation (Laskowski, Moiron, and Niemela 2021) in line with this framework. Currently, little is known about the extent to which correlations between different behaviours are negative, as expected due to trade‐offs between them (i.e., variation in resource allocation), or whether such correlations are positive, as expected due to heterogeneity in individual condition or quality (i.e., variation in resource acquisition). As explained above, the existence of trade‐offs between behaviours is a fundamental assumption in behavioural ecology (Stearns 1977, 1989) but so is the assumption of heterogeneity in individual condition or quality. For example, heterogeneity in individual condition or quality is key to concepts such as honest signalling (Zahavi 1975). Here, we use parental care as a case study for exploring whether the observed variation in parents' behaviour is mostly driven by variation in resource acquisition or resource allocation.

Parental care is defined as any parental trait that increases offspring fitness, often at a cost to the parent's own fitness (Smiseth, Kölliker, and Royle 2012). There is remarkable variation within and among species in the amount, type and duration of care provided by male and female parents (Clutton‐Brock 1991; Royle, Smiseth, and Kölliker 2012). Traditionally, such variation has been attributed to differences in the benefits and costs of care driven by the type and harshness of environmental stresses to which offspring are exposed (e.g., predation, cannibalism, disease and starvation), the extent to which parents can neutralise these stresses and the fitness costs that parents incur from providing care (Clutton‐Brock 1991; Royle, Smiseth, and Kölliker 2012). Although there is general agreement that variation in the benefits and costs of care is an important determinant of variation in parental care (Smiseth 2018), such variation often persists when parents provide care under standardised laboratory conditions where the benefits and costs should be similar for all individuals (e.g., Andrews, Kruuk, and Smiseth 2017). Thus, there must be other sources of variation in parental care, including individual differences in life‐history strategies with respect to how parents allocate their time and energy budgets towards care.

In species with elaborate parental care, where care involves suites of traits, we can assess the relative contribution of variation in resource allocation and resource acquisition by examining relationships between parental behaviours (Andrews, Kruuk, and Smiseth 2017). Prior work has interpreted negative relationships between parental behaviours as evidence of trade‐offs between parental behaviours and positive relationships as evidence of individual variation in parental quality (Andrews, Kruuk, and Smiseth 2017). However, this interpretation ignores potential effects of trade‐offs *between* breeding attempts, which would generate positive relationships between parental behaviours if individuals vary in how they allocate to current reproduction at the expense of future reproduction. Thus, positive relationships between parental behaviours may reflect variation in resource acquisition *or* variation in resource allocation between current and future reproduction. Separating between these two sources of variation requires additional information on allocation towards future reproduction, such as data on post‐breeding survival. In line with the predictions set out by Laskowski, Moiron, and Niemela (2021), we would thus expect negative relationships between parental behaviours and post‐breeding survival if there is variation in resource allocation between breeding attempts and positive relationships if there is variation in resource acquisition.

Prior work on the relationship between parental behaviours has provided mixed evidence, with some studies reporting a negative relationship (Rytkönen et al. 1995; Lissåker and Kvarnemo 2006; Walling et al. 2008) and others reporting a positive one (Spencer 2005; Kopisch, Schwagmeyer, and Mock 2005; Andrews, Kruuk, and Smiseth 2017). There are several potential explanations for these inconsistent results. Firstly, there may be differences between species with respect to the relative amount of variation in resource allocation and resource acquisition. This is unlikely to be the only explanation, given that there is mixed evidence from studies on the same species. For example, one study on the burying beetle *Nicrophorus vespilloides* reported a negative relationship (Walling et al. 2008), while another reported a positive relationship (Andrews, Kruuk, and Smiseth 2017). Secondly, there might be sex differences in the relationship between parental behaviours, given that males and females often differ in their contributions towards care in species with biparental care (Whittingham 1989; Lavery and Keenleyside 1990; Margulis 1998; Kokko and Jennions 2012). Finally, there might be different relationships between parental behaviours when parents provide care on their own or with a partner (Wolf, Ketterson, and Nolan Jr. 1990; Lavery and Reebs 1994; Markman, Yom‐Tov, and Wright 1996; Hunt and Simmons 2002).

The burying beetle *N. vespilloides* is an excellent study system for disentangling these effects and advancing our understanding of how variation in resource allocation versus resource acquisition drives intraspecific variation in parental care. This species breeds on carcasses of small vertebrates, and both parents often engage in elaborate care, comprising direct care (provisioning food or interacting with the larvae) and indirect care (guarding or carcass maintenance). At least one of the parents, usually the female, stays with the larvae until they disperse from the carcass, which occurs 4–7 days after hatching. There is variation in both sexes with respect to time spent on different parental behaviours (Andrews, Kruuk, and Smiseth 2017) and the duration of parental care (Ford and Smiseth 2016). Furthermore, there are pronounced sex differences with females spending more time on direct and indirect care (Smiseth and Moore 2004; Georgiou‐Shippi, Paquet, and Smiseth 2018) and caring for longer than males (Ford and Smiseth 2016). Finally, parents may provide care on their own or with a partner (Smiseth et al. 2005; Pilakouta, Hanlon, and Smiseth 2018), and prior work has shown that parents adjust their parental behaviour depending on the presence or absence of a partner (Smiseth et al. 2005; Pilakouta, Hanlon, and Smiseth 2018).

Our study focuses on three main questions. Firstly, how do individual differences in life‐history strategies drive variation in parental care? If the main driver is individual differences in resource allocation within breeding attempts, we expect negative relationships between parental behaviours but no relationship between parental care and post‐breeding lifespan. If the main driver is differences in resource allocation between breeding attempts, we expect positive relationships between parental behaviours but negative relationships between parental care and post‐breeding lifespan. If the main driver is differences in resource acquisition among individuals, we expect positive relationships between parental behaviours, as well as between parental care and post‐breeding lifespan. Secondly, do relationships between parental behaviours and between parental care and post‐breeding lifespan differ between males and females? We expect that variation in resource allocation or acquisition has a stronger effect on female parents, given that they are working closer to their maximum capacity than males in this species (Smiseth and Moore 2004). Thirdly, do relationships between parental behaviours differ under uniparental vs. biparental care? We expect weaker relationships when parents provide care with a partner, because parental decisions are also influenced by their partner's contribution.

## 2 Materials and Methods

Our study takes advantage of an existing dataset (Pilakouta, Hanlon, and Smiseth 2018) to address a new research question. Below we provide an overview of the experimental design that was used to generate these data.

### 2.1 Experimental Design

We used fifth‐ and sixth‐generation beetles from our outbred laboratory population, which was maintained at 21°C and a 16 h:8 h light: dark cycle. This population derived from beetles originally collected in the wild in Edinburgh, UK. Each adult was housed in a transparent plastic container (12 × 8 × 2 cm) filled with moist soil. All adult beetles were fed raw organic beef *ad libitum* twice a week before and after breeding.

Our experimental design consisted of a biparental care treatment, where male and female parents provided care together, and a uniparental care treatment, where parents provided care on their own (Figure S1). We kept carcass mass and number of offspring per parent constant between treatments to avoid confounding effects due to differences in resource availability or parental workload, respectively (Figure S1). We mated virgin males and females after they reached sexual maturity, 10 days after eclosion. We ensured that mated pairs shared no common ancestor for at least two generations to avoid inbreeding. We placed each pair in a transparent plastic container (17 × 12 × 6 cm) filled with 1 cm of moist soil. We provided each pair with two defrosted mouse carcasses (11 ± 1 g) to initiate breeding. We randomly assigned half of the pairs to the biparental care treatment (*n* = 65) and the other half to the uniparental care treatment (*n* = 65). After eggs had been laid, we moved the parents and the carcasses to fresh containers with moist soil, so that the larvae would hatch in isolation from the parents. In the biparental care treatment, the two parents were transferred to the same container along with both carcasses. Meanwhile, in the uniparental care treatment, males and females were transferred to separate containers, and each parent was given one of the two carcasses. We left the eggs in the original container, using them later to generate our experimental broods.

Once these eggs started hatching, we collected the newly hatched larvae, using them to create experimental broods for the uniparental care (15 larvae) and biparental care (30 larvae) treatments (Figure S1). We always used larvae of mixed parentage when generating experimental broods to exclude potential confounding effects due to parent‐offspring coadaptation. Parents do not discriminate between their own broods and unrelated foster broods provided that the larvae are at a similar developmental stage (Oldekop et al. 2007). Parents kill any larvae that arrive on the carcass too early (Müller and Eggert 1990), so we only allocated parents with an experimental brood once their own eggs had started to hatch. Because we were limited by the number of larvae that hatched at the same time, our final sample sizes were *n* = 40 for the biparental care treatment and *n* = 49 for the uniparental care treatment.

### 2.2 Amount of Care, Duration of Care and Post‐Breeding Lifespan

We performed behavioural observations of parental care 24 h after the parents were given a brood. We did the observations at this stage in larval development because it corresponds to a peak in post‐hatching care (Smiseth, Darwell, and Moore 2003). We used instantaneous sampling every 1 min for 30 min, recording the number of scans that each parent provided direct care, defined as provisioning food to larvae or interacting with them, and indirect care, defined as guarding the brood or maintaining the carcass.

We also determined the overall duration of care by checking the containers twice a day and recording whether the parents were present on the carcass. Any parent that was absent from the carcass on more than two consecutive occasions was deemed to have deserted the brood. Such parents were removed from the containers to prevent them from committing infanticide (Pilakouta, Hanlon, and Smiseth 2018). We used the last observation when the parent was present on the carcass to estimate its duration of care.

Lastly, we measured post‐breeding lifespan for each parent to investigate relationships between parental care and allocation towards current and future reproduction. At the end of the breeding attempt, we transferred each parent to separate containers (12 × 8 × 2 cm) filled with moist soil. We monitored each individual twice a week until death to determine its post‐breeding lifespan as a proxy for somatic investment and allocation to future reproduction (Pilakouta et al. 2016).

### 2.3 Data Analysis

We performed all analyses in R version 4.4.0 (R Development Core Team 2024). We used the ‘ggplot2’ package to generate figures (Wickham 2009). Because all our response variables were discrete traits, we used generalised linear models fitted with a negative binomial error distribution (lifespan) or a quasipoisson error distribution (amount of care and duration of care). The quasipoisson distribution was used to account for overdispersion in our data by including a dispersion parameter that describes additional variance. Given that male and female parents were part of the same pair and were therefore not independent, our intention was to use mixed‐effects models with ‘pair ID’ as a random effect. However, this was not possible with this particular dataset because the mixed‐effects models did not achieve convergence.

To test for a trade‐off between different components of parental care, we used a model with indirect care as the response variable and direct care as the main explanatory variable. We included sex (male or female), number of parents (one or both parents), direct care × number of parents, direct care × sex and sex × number of parents as additional factors in this model. To examine the relationship between the amount and duration of care, we used a model with care duration as the response variable and total amount of care (sum of direct and indirect care) as the main explanatory variable. Sex, number of parents, total care × number of parents, total care × sex and sex × number of parents were also included as additional factors in this model. Next, we examined the relationship between parental care and post‐breeding lifespan using four models: (i) a model with lifespan as the response variable, total amount of care as the main explanatory variable, as well as sex, number of parents, total amount of care × sex, total amount of care × number of parents and sex × number of parents, (ii) a model with lifespan as the response variable, duration of care as the main explanatory variable, as well as sex, number of parents, duration of care × sex, duration of care × number of parents and sex × number of parents, (iii) a model with lifespan as the response variable, amount of direct care as the main explanatory variable, as well as sex, number of parents, direct care × sex, direct care × number of parents and sex × number of parents and (iv) a model with lifespan as the response variable, amount of indirect care as the main explanatory variables, as well as sex, number of parents, indirect care × sex, indirect care × number of parents and sex × number of parents. Lastly, we examined the relationship between post‐breeding lifespan and offspring fitness (as a measure of parental quality) using two additional models: (i) a model with lifespan as the response variable, offspring size (i.e., average larval mass) as the main explanatory variable, as well as sex, number of parents, offspring size × sex, offspring size × number of parents and sex × number of parents and (ii) a model with lifespan as the response variable, offspring number (i.e., brood size at the dispersal stage) as the main explanatory variable, as well as sex, number of parents, offspring number × sex, offspring number × number of parents and sex × number of parents.

Each of these eight statistical models was run both with and without body size (i.e., pronotum width) as a covariate. The rationale for this was that if body size is an important contributor to resource acquisition, we would expect that any positive relationships between parental behaviours or between parental care and lifespan would be weaker or absent when body size is taken into account. Although body size can reflect differences in resource acquisition at the larval stage (Pilakouta et al. 2016), we note that it is just one potential source of variation in resource acquisition. There are likely to be other factors contributing to variation in resource acquisition that we have not measured in this study.

## 3 Results

### 3.1 How Does Variation in Life‐History Strategies Drive Variation in Parental Care?

**FIGURE 1:**
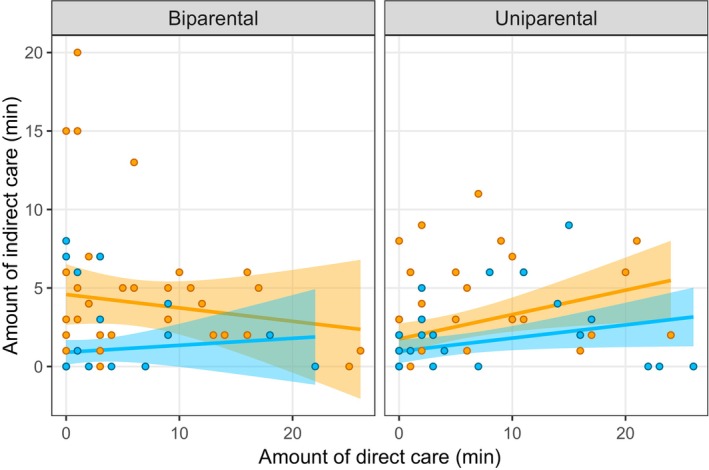
Amount of direct versus indirect care provided by males (blue) and females (orange) 24 h after hatching in the uniparental (*n* = 49) and biparental (*n* = 40) treatments. Direct care includes food provisioning and interactions with larvae, whereas indirect care includes carcass maintenance and guarding. The shaded area around the line of best fit indicates the 95% confidence interval.

**FIGURE 2:**
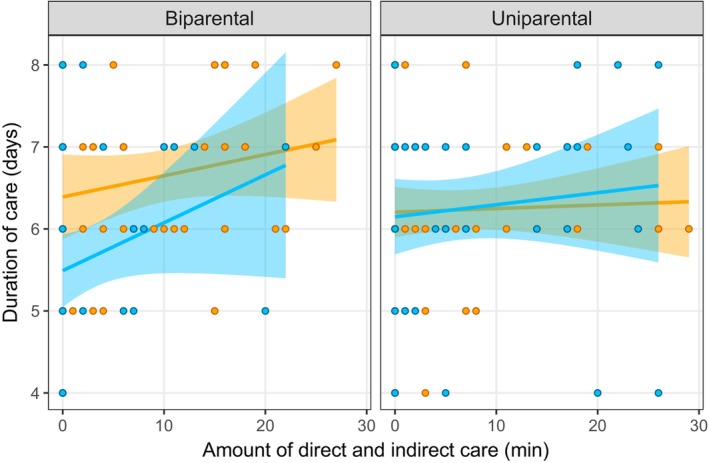
Amount of total care (sum of direct and indirect care) versus duration of care by males (blue) and females (orange) in the uniparental (*n* = 49) and biparental (*n* = 40) treatments. The amount of care provided by parents was measured during 30‐min behavioural observations done 24 h after hatching. Duration of care refers to the number of days each parent was present on the carcass before deserting the brood. The shaded area around the line of best fit indicates the 95% confidence interval.

We first examined the relationship between different types of parental behaviours (direct vs. indirect care). There was a statistically significant interaction between direct care and the number of parents present (Table 1), suggesting a stronger positive relationship between direct and indirect care in the uniparental than the biparental treatment (Figure 1). There was also a marginally nonsignificant positive relationship between the total amount of care provided during the behavioural observation (24 h after hatching) and the duration of care in days (Table 1 and Figure 2). Thus, there was no evidence for a trade‐off between the amount of time spent providing direct and indirect care provided by parents and no evidence that those parents that provided more care deserted their brood sooner.

**TABLE 1:** Results of statistical models examining the relationship between different parental behaviours, between parental care and lifespan and between offspring fitness (as a measure of parental quality) and lifespan. Response variables are italicised. LR denotes the likelihood ratio. Estimates refer to standardised regression coefficients calculated using the R package ‘effectsize’ (Ben‐Sachar and Lüdecke 2020). The reference categories for these models are ‘female’ for sex and ‘biparental’ for number of parents. Statistically significant results (*p* < 0.05) are indicated in bold and marginally nonsignificant results (0.05 < *p* < 0.10) are indicated in italics.

	Estimate	LR χ^2^	*p*
*Indirect care*			
Direct care	−0.11	2.55	0.11
Sex	−1.44	20.5	**< 0.0001**
Number of parents	−0.59	2.45	0.12
Direct care × Number of parents	0.42	4.28	**0.039**
Direct care × Sex	0.08	0.13	0.72
Sex × Number of parents	0.78	2.35	0.13
*Duration of care*			
Total care amount	0.04	3.14	*0.077*
Sex	−0.12	2.79	*0.09*
Number of parents	−0.05	0.10	0.75
Total care amount × Number of parents	−0.04	1.77	0.18
Total care amount × Sex	0.02	0.75	0.38
Sex × Number of parents	0.12	4.23	**0.040**
*Post‐breeding lifespan*			
Total care amount	−0.07	0.32	0.57
Sex	−0.05	3.06	*0.08*
Number of parents	0.01	0.01	0.92
Total care amount × Number of parents	0.04	0.71	0.40
Total care amount × Sex	0.07	2.22	0.14
Sex × Number of parents	−0.04	0.16	0.69
*Post‐breeding lifespan*			
Duration of care	0.02	4.46	**0.035**
Sex	0.02	0.88	0.35
Number of parents	0.05	0.01	0.93
Care duration × Number of parents	−0.03	0.36	0.55
Care duration × Sex	0.06	1.60	0.21
Sex × Number of parents	−0.11	1.62	0.20
*Post‐breeding lifespan*			
Direct care	−0.07	0.19	0.67
Sex	−0.03	2.69	0.10
Number of parents	0.03	0.01	0.91
Direct care × Number of parents	0.03	0.29	0.59
Direct care × Sex	0.09	4.31	**0.038**
Sex × Number of parents	−0.06	0.56	0.46
*Post‐breeding lifespan*			
Indirect care	< 0.01	0.22	0.64
Sex	−0.04	2.12	0.15
Number of parents	0.04	0.09	0.77
Indirect care × Number of parents	0.01	0.06	0.81
Indirect care × Sex	−0.06	1.23	0.27
Sex × Number of parents	−0.05	0.37	0.55
*Post‐breeding lifespan*			
Offspring size	< 0.01	1.19	0.28
Sex	−0.02	1.98	0.16
Number of parents	0.04	< 0.01	0.98
Offspring size × Number of parents	< 0.01	0.01	0.93
Offspring size × Sex	−0.06	1.81	0.18
Sex × Number of parents	−0.09	0.99	0.32
*Post‐breeding lifespan*			
Offspring number	0.08	2.06	0.15
Sex	−0.03	1.68	0.19
Number of parents	0.03	0.02	0.88
Offspring number × Number of parents	−0.08	3.35	*0.067*
Offspring number × Sex	< 0.01	0.05	0.82
Sex × Number of parents	−0.05	0.34	0.56

**FIGURE 3:**
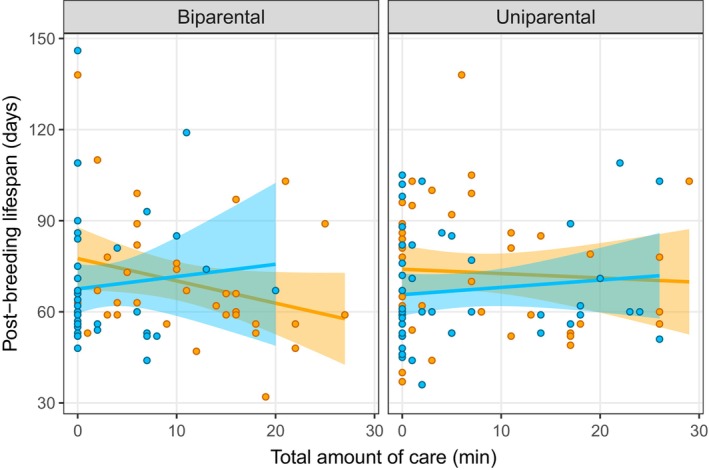
Relationship between the total amount of care and the parents' post‐breeding lifespan (males: blue, females: orange) in the uniparental (n = 49) and biparental (n = 40) treatments. Total amount of care refers the amount of direct and indirect care provided by parents during 30‐min behavioural observations done 24 h after hatching. The shaded area around the line of best fit indicates the 95% confidence interval.

**FIGURE 4:**
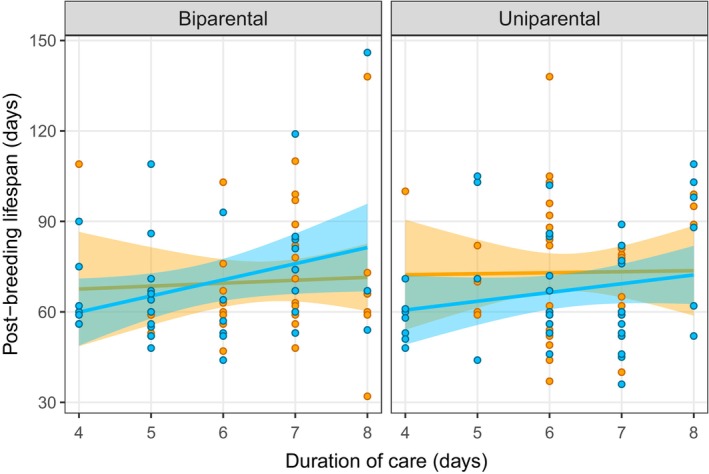
Relationship between duration of care and the parents' post‐breeding lifespan (males: blue, females: orange) in the uniparental (n = 49) and biparental (n = 40) treatments. Duration of care refers to the number of days each parent was present on the carcass before deserting the brood. The shaded area around the line of best fit indicates the 95% confidence interval.

We then examined whether post‐breeding lifespan was related to different measures of parental effort (amount of indirect care, amount of direct care, total amount of care and duration of care) or offspring fitness traits as measures of parental quality (average larval mass at dispersal and brood size at dispersal). There was no relationship between post‐breeding lifespan and indirect care or the total amount of care (Table 1 and Figure 3), but there was a significant positive relationship between post‐breeding lifespan and duration of care (Table 1 and Figure 4). There was also a significant interaction between parent sex and direct care on lifespan, indicating a stronger positive relationship between direct care and lifespan for males than for females (Table 1). These results suggest that parents that provide more parental care do not experience a trade‐off of reduced lifespan after the breeding attempt, potentially due to variation in prior resource acquisition. Lastly, there was no evidence for a relationship between offspring fitness traits and the parents' post‐breeding lifespan (Table 1).

### 3.2 Do These Relationships Differ Between Males and Females?

We next assessed whether relationships between parental behaviours, and between parental care and post‐breeding lifespan, were different for males and females. There was very limited evidence for such a pattern. The only exception was with regard to the relationship between direct care and post‐breeding lifespan, where we found a stronger positive relationship for males than females (Table 1 and Figures 1, 2, 3, 4). Thus, there was little support for our prediction that the relationship between different components of care would be stronger in females than in males.

### 3.3 Do These Relationships Differ Between Biparental and Uniparental Care?

Finally, we asked whether relationships between parental behaviours, and between parental care and post‐breeding lifespan, differed between parents providing care with a partner (biparental care) and those providing care on their own (uniparental care). As mentioned above, we found a stronger positive relationship between direct and indirect care in the uniparental than the biparental treatment (Figure 1). There was also a marginally nonsignificant interaction suggesting a positive relationship between offspring number and lifespan in the biparental treatment but not in the uniparental treatment. The relationship between the duration and amount of care or between parental care and post‐breeding lifespan did not differ between the uniparental and biparental treatments (Table 1 and Figures 3 and 4). Thus, there was little support for our prediction of a weaker relationship between parental behaviours when parents provided care with a partner.

### 3.4 Do These Relationships Become Weaker When Parent Body Size Is Taken Into Account?

If body size is an important contributor to variation in resource acquisition, we would expect that any positive relationships between parental behaviours or between parental care and lifespan would be weaker or absent when body size is taken into account. Our results on the relationship between direct and indirect are, between the amount and duration of care, between indirect care and lifespan and between total amount of care and lifespan were qualitatively similar, regardless of whether body size was included as a covariate (Table 1 and Table S1). Nevertheless, the statistically significant relationships between duration of care and lifespan and between direct care and lifespan (Table 1) became marginally nonsignificant (*p* = 0.075 and *p* = 0.090, respectively) when body size was included in the model (Table S1). The opposite pattern was seen in the effect of interactions between offspring size × sex and offspring number × number of parents on lifespan, which were marginally nonsignificant in the models excluding body size but became statistically significant when body size was included (Table 1 and Table S1). Thus, taking body size into account in our statistical models did not have a consistent effect on the strength of the relationship between these life‐history traits.

## 4 Discussion

Our aim was to integrate the study of parental care more generally into life‐history theory to examine whether relationships between parental behaviours are mainly driven by variation in resource allocation or resource acquisition. We found no support for the idea that variation in parental care was driven by individual differences in resource allocation within or between breeding attempts, as there was no evidence for a negative correlation between parental behaviours or between parental care and post‐breeding lifespan, which would be indicative of a trade‐off between them. Instead, we found some positive relationships between parental behaviours. For example, parents that spent more time providing direct care also provided more indirect care (in the uniparental but not in the biparental treatment). There was also some evidence that those parents that spent more time providing care 24 h after hatching also provided care for longer. In addition, parents that provided more parental care did not experience a trade‐off of reduced lifespan after the breeding attempt. In fact, there was a positive relationship between the duration of care provided and the parents' post‐breeding lifespan. Our findings suggest that variation in parental care is, at least to some extent, driven by individual differences in resource acquisition associated with heterogeneity among individuals or individual differences in quality. This result is in line with a recent meta‐analysis, which revealed that behaviours associated with higher reproductive success tend to also be associated with increased survival, suggesting that variation in behaviour among individuals reflects differences in resource acquisition (Haave‐Audet et al. 2022).

Our results have conceptual implications for applying the framework of van Noordwijk and de Jong (1986) to how individual differences in life‐history strategies might drive within‐species variation in parental care. Prior work has considered variation in resource allocation and variation in resource acquisition (Andrews, Kruuk, and Smiseth 2017) but failed to partition variation in resource allocation into two components: resource allocation *within* and *between* breeding attempts. This is an important distinction because positive relationships between parental behaviours may reflect variation in resource acquisition, as argued by Andrews, Kruuk, and Smiseth (2017), *or* they may reflect variation in resource allocation between breeding attempts. We can separate between these two alternatives by including data on post‐breeding lifespan, as we did here, or on other components of allocation towards future survival and reproduction (Laskowski, Moiron, and Niemela 2021).

We use the term individual differences in resource acquisition to place our work within the conceptual framework of van Noordwijk and de Jong (1986), but we emphasise that we make no assumptions about the nature or origin of these individual differences. As originally proposed by van Noordwijk and de Jong (1986), there may be differences with respect to the amount of resources that individuals have acquired, either during development or as adults, or there may even be differences in how efficiently individuals turn acquired resources into various life‐history functions. Here, we explored body size as one potential source of heterogeneity in resource acquisition. As expected, when we included body size as a covariate in our models, this weakened some of the positive correlations; that is, between duration of care and lifespan and between direct care and lifespan. However, we note that body size was negatively correlated with post‐breeding lifespan; that is, smaller individuals lived for longer than lifespan. Thus, any effects of body size were not driven by larger individuals living for longer and providing more care as we expected. Instead, body size may be linked to some unknown source of heterogeneity among individuals, such as their efficiency in turning acquired resources into key life‐history functions. Understanding the origins and nature of such heterogeneity among individuals, whether due to phenotypic or genetic differences between individuals, is now a major priority in this field.

We suggest that, in populations where variation in parental care is largely driven by individual differences in resource acquisition or parental quality, we should expect positive relationships between parental behaviours. Under this scenario, it would not matter which parental behaviours we measure, as high‐quality individuals should allocate more time and energy towards all forms of care. In contrast, in populations where variation in parental care is largely driven by variation in resource allocation, we expect trade‐offs between different parental behaviours, so individuals that spend more time on one behaviour should spend less time on another behaviour or less time on the same behaviour at a different time point. In other words, different individuals would provide the highest amount of care depending on which parental behaviours we measured and when we measured them. We encourage other researchers to apply this approach to more study systems with elaborate parental care where it is possible to measure multiple parental behaviours and post‐breeding survival, such as birds and fishes. This will enable researchers to choose the most appropriate methodology in terms of which parental behaviours to record and whether to record such behaviours across multiple timescales.

Both formal theory and verbal arguments for the evolution of parental care are based on the fundamental assumption of life‐history trade‐offs, either between different parental behaviours within a breeding attempt or between parental care and future survival or reproduction (Clutton‐Brock 1991; Smiseth, Kölliker, and Royle 2012). Such trade‐offs are expected because parents will have a fixed time and energy budget that they can allocate towards different functions, such as specific parental behaviours. Our results should not be interpreted as evidence against the existence of such trade‐offs. Instead, our results highlight that in some species or populations, trade‐offs between different parental behaviours or between parental care and post‐breeding survival can be masked by heterogeneity among parents, for example, due to individual differences in quality. Even within populations, seasonal or annual variation in resource availability will influence the level of resource acquisition by individuals in the population, thus affecting variation in individual quality and the manifestation of these life‐history trade‐offs.

Our second main question was whether the relationships between parental behaviours were stronger in females than in males. We expected relationships between parental behaviours to be stronger in females because *N. vespilloides* females spend substantially more time providing care than males and are working closer to their maximum capacity (Smiseth and Moore 2004; Ford and Smiseth 2016; Pilakouta, Hanlon, and Smiseth 2018; Pilakouta et al. 2023). Nevertheless, we found no evidence for this prediction. Our results suggest that variation in resource acquisition, or individual quality, had a similar effect on male and female behaviour. This raises the question of why variation in male quality may determine male contributions towards care, even though males work below their maximum capacity (Smiseth et al. 2005). This may reflect that lower‐quality males desert the broods earlier to pursue other breeding opportunities. This seems unlikely given that lower‐quality males would presumably benefit less from deserting because they should be less successful in securing a second breeding attempt than higher‐quality males. Alternatively, our results may reflect that females are less tolerant towards lower‐quality males, driving them away from the carcass at an earlier stage. There is evidence for sexual conflict over consumption of the breeding carcass in our study species (Pilakouta, Richardson, and Smiseth 2016). Thus, the duration of male care may be partially under the female's control, in which case our results may reflect that females drive away low‐quality males from the carcass earlier but allow high‐quality males to stay for longer.

Our third main question was whether the relationships between different parental behaviours were weaker when parents provided care with a partner (biparental care) than when they provided care on their own (uniparental care). We expected weaker relationships in the former scenario because the parents' decisions about how much care to provide would also be influenced by their partner's behaviour and negotiation within the pair (Smiseth and Moore 2004; Pilakouta, Richardson, and Smiseth 2015; Ford and Smiseth 2016). However, we found little support for this prediction. Although there was a stronger positive relationship between direct and indirect care in the uniparental treatment, there was no such pattern when examining relationships between other parental behaviours or parental care and lifespan. One possible explanation is that negotiation is not the only mechanism determining contributions to care by male and female parents. Prior work on *N. vespilloides* shows that parents also adjust their behaviour based on their own state and the state of their partner (Pilakouta, Richardson, and Smiseth 2015). Some of these mechanisms may weaken relationships between parental behaviours, and others may strengthen them. For example, relationships between parental behaviours may be strengthened during biparental care if high‐ and low‐quality individuals differ in how they respond to the quality of their partner.

Our study adds to prior work on life‐history trade‐offs and effects of individual variation in resource acquisition on such trade‐offs in the burying beetle *Nicrophorus vespilloides* . There is mixed evidence for trade‐offs between life‐history traits and between behaviours. For example, there is good evidence for a trade‐off between the number and size of offspring (e.g., Monteith, Andrews, and Smiseth 2012; Andrews, Kruuk, and Smiseth 2017), although the magnitude of this trade‐off depends on resource acquisition prior to breeding (i.e., carcass size) (Smiseth et al. 2014). Meanwhile, there is mixed evidence for trade‐offs between parental behaviours, with one study reporting a negative relationship (Walling et al. 2008) and another reporting a positive relationship (Andrews, Kruuk, and Smiseth 2017). This species has attracted interest as a system for studying how individual variation in resource acquisition masks life‐history trade‐offs, given that it is relatively straightforward to experimentally control and manipulate resource acquisition at different stages of the life cycle. For example, Richardson and Smiseth (2020) manipulated resource acquisition at three different stages of the life cycle: larval development (i.e., adult body size), nutritional state (i.e., adult body mass at onset of breeding) and resource acquisition prior to breeding (i.e., carcass size). This study found no evidence of a life‐history trade‐off between the number and size of offspring when information on individual variation in resource acquisition was excluded from the model. However, there was evidence for such a life‐history trade‐off when information on individual variation in resource acquisition was included in the model. This trade‐off was influenced by variation in resource acquisition prior to breeding (i.e., carcass size), as there was a significant negative relationship between the number and size of offspring when breeding on smaller carcasses but not when breeding on larger carcasses. There was no evidence for a trade‐off between brood mass and post‐breeding lifespan (used as proxies for current and future reproduction) regardless of whether information on individual variation in resource acquisition was excluded from or included in the model. These findings support our conclusion that relationship life‐history traits are largely driven by individual differences in resource acquisition rather than individual differences in resource allocation.

In conclusion, we found that parents that spent more time providing direct care also provided more indirect care (in the uniparental treatment), parents that spent more time providing care 24 h after hatching tended to provide care for longer, and parents that provided care for longer also had a longer post‐breeding lifespan. This suggests that variation in care was mainly driven by individual differences in resource acquisition (i.e., parental quality) rather than individual differences in resource allocation. Our findings therefore suggest that intraspecific variation in parental quality can mask reproductive investment trade‐offs within populations.

## Author Contributions

**Natalie Pilakouta:** conceptualization (equal), data curation (lead), formal analysis (lead), methodology (equal), supervision (equal), visualization (lead), writing – original draft (equal), writing – review and editing (lead). **Elizabeth J. H. Hanlon:** data curation (supporting), investigation (lead), writing – review and editing (supporting). **Per T. Smiseth:** conceptualization (equal), funding acquisition (lead), methodology (equal), project administration (lead), resources (lead), supervision (equal), writing – original draft (equal), writing – review and editing (equal).

## Conflicts of Interest

The authors declare no conflicts of interest.

## Supporting information


Data S1.

Figure S1.

Table S1.

## Data Availability

Our study uses an already published dataset, which can be found on the Dryad Digital Repository: https://datadryad.org/stash/dataset/doi:10.5061/dryad.s7n04j8.
