# Supplementary material for: Intraspecific Variation in Parental Care May Reflect Variation in Parental Quality
Source: Ecol Evol. 2024 Nov 13;14(11):e70578. doi: 10.1002/ece3.70578 (PMC11560317; doi:10.1002/ece3.70578)
Supplement: Supplementary file 2 — Figure S1. [file ECE3-14-e70578-s003.pdf]

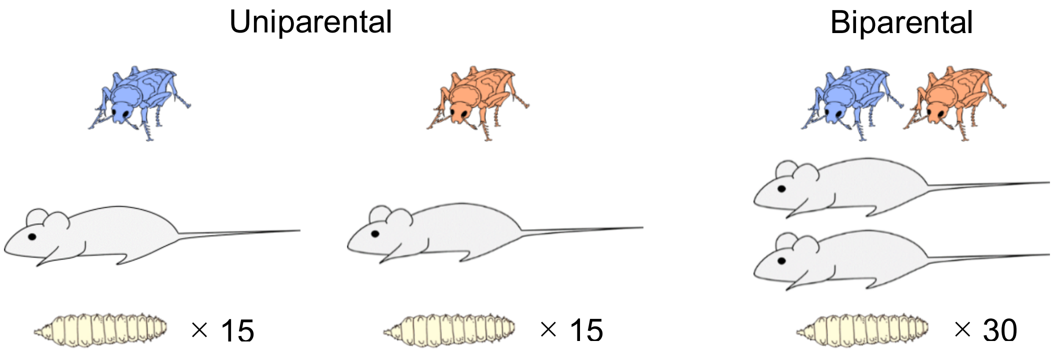

Supplementary Figure 1. Experimental design illustration (not drawn to scale). Males (blue) and females (orange) in the uniparental treatment were separated after egg laying and were each provided with one mouse of a standardized size (10–12g) and a mixed-parentage brood of 15 larvae. Parents in the biparental treatment were allowed to stay together and were provided with two mice of a standardized size (10–12g) and a mixed-parentage brood of 30 larvae.
