# Supplementary material for: Intraspecific Variation in Parental Care May Reflect Variation in Parental Quality
Source: Ecol Evol. 2024 Nov 13;14(11):e70578. doi: 10.1002/ece3.70578 (PMC11560317; doi:10.1002/ece3.70578)
Supplement: Supplementary file 3 — Table S1. [file ECE3-14-e70578-s001.docx]

**Supplementary Table 1.** Results of statistical models examining the relationship between different parental behaviours, between parental care and lifespan, and between offspring fitness (as a measure of parental quality) and lifespan. Response variables are italicised. LR denotes likelihood ratio. Estimates refer to standardised regression coefficients calculated using the R package ‘effectsize’ (Ben-Sachar et al. 2020). The reference categories for these models are “female” for sex and “biparental” for number of parents. Pronotum width was used as a proxy for body size. Statistically significant results are indicated in bold.

| *Indirect care* | Estimate | LR $\chi^{2}$ | *P* |
| --- | --- | --- | --- |
| Direct care | -0.13 | 2.39 | 0.12 |
| Sex | -1.48 | 20.4 | **<0.001** |
| Number of parents | -0.64 | 2.76 | 0.10 |
| Pronotum | 0.14 | 1.60 | 0.21 |
| Direct care × Number of parents | 0.42 | 4.26 | **0.039** |
| Direct care × Sex | 0.12 | 0.33 | 0.57 |
| Sex × Number of parents | 0.87 | 2.83 | 0.093 |
|  |  |  |  |
| *Duration of care* | Estimate | LR $\chi^{2}$ | *P* |
| Total care amount | 0.04 | 3.27 | *0.07* |
| Sex | -0.11 | 2.98 | *0.08* |
| Number of parents | -0.04 | 0.13 | 0.72 |
| Pronotum | -0.01 | 1.19 | 0.28 |
| Total care amount × Number of parents | -0.04 | 1.61 | 0.20 |
| Total care amount × Sex | 0.02 | 0.50 | 0.48 |
| Sex × Number of parents | 0.11 | 3.57 | *0.059* |
|  |  |  |  |
| *Post-breeding lifespan* | Estimate | LR $\chi^{2}$ | *P* |
| Total care amount | -0.06 | 0.10 | 0.75 |
| Sex | -0.02 | 3.59 | *0.058* |
| Number of parents | 0.05 | <0.01 | 0.98 |
| Pronotum | -0.09 | 21.4 | <0.001 |
| Total care amount × Number of parents | 0.05 | 1.04 | 0.31 |
| Total care amount × Sex | 0.05 | 1.32 | 0.25 |
| Sex × Number of parents | -0.10 | 1.24 | 0.27 |
|  |  |  |  |
| *Post-breeding lifespan* | Estimate | LR $\chi^{2}$ | *P* |
| Duration of care | <0.01 | 3.18 | *0.075* |
| Sex | 0.04 | 1.37 | 0.24 |
| Number of parents | 0.07 | 0.01 | 0.94 |
| Pronotum | -0.09 | 21.4 | <0.001 |
| Care duration × Number of parents | -0.02 | 0.14 | 0.71 |
| Care duration × Sex | 0.06 | 2.11 | 0.15 |
| Sex × Number of parents | -0.16 | 3.67 | *0.056* |
|  |  |  |  |
| *Post-breeding lifespan* | Estimate | LR $\chi^{2}$ | *P* |
| Direct care | -0.05 | 0.09 | 0.77 |
| Sex | <0.01 | 2.94 | *0.087* |
| Number of parents | 0.06 | <0.01 | 0.99 |
| Pronotum | -0.09 | 20.2 | **<0.001** |
| Direct care × Number of parents | 0.02 | 0.25 | 0.62 |
| Direct care × Sex | 0.07 | 2.87 | 0.09 |
| Sex × Number of parents | -0.12 | 2.17 | 0.14 |
|  |  |  |  |
| *Post-breeding lifespan* | Estimate | LR $\chi^{2}$ | *P* |
| Indirect care | <0.01 | 0.02 | 0.88 |
| Sex | -0.01 | 2.28 | 0.13 |
| Number of parents | 0.07 | 0.12 | 0.73 |
| Pronotum | -0.10 | 22.6 | **<0.001** |
| Indirect care × Number of parents | 0.04 | 1.00 | 0.32 |
| Indirect care × Sex | -0.05 | 0.89 | 0.35 |
| Sex × Number of parents | -0.10 | 1.47 | 0.23 |
|  |  |  |  |
| *Post-breeding lifespan* | Estimate | LR $\chi^{2}$ | *P* |
| Offspring size | 0.03 | 0.10 | 0.75 |
| Sex | 0.02 | 1.98 | 0.16 |
| Number of parents | 0.08 | 0.01 | 0.91 |
| Pronotum | -0.10 | 23.5 | **<0.001** |
| Offspring size × Number of parents | <0.01 | <0.01 | >0.99 |
| Offspring size × Sex | -0.08 | 4.24 | **0.039** |
| Sex × Number of parents | -0.15 | 3.48 | *0.062* |
|  |  |  |  |
| *Post-breeding lifespan* | Estimate | LR $\chi^{2}$ | *P* |
| Offspring number | 0.08 | 1.15 | 0.28 |
| Sex | <0.01 | 1.91 | 0.17 |
| Number of parents | 0.06 | 0.03 | 0.85 |
| Pronotum | -0.10 | 23.8 | **<0.001** |
| Offspring number × Number of parents | -0.09 | 4.96 | **0.026** |
| Offspring number × Sex | -0.02 | 0.27 | 0.60 |
| -Sex × Number of parents | -0.11 | 2.00 | 0.16 |
